# Ophthalmic Diagnosis and Novel Management of Infantile Refsum Disease with Combination Docosahexaenoic Acid and Cholic Acid

**DOI:** 10.1155/2021/1345937

**Published:** 2021-10-09

**Authors:** Omar Elghawy, Alice Y. Zhang, Ryan Duong, William G. Wilson, Eugene Y. Shildkrot

**Affiliations:** ^1^University of Virginia School of Medicine, Charlottesville, Virginia, USA; ^2^UVA Health Department of Ophthalmology, Charlottesville, Virginia, USA; ^3^UVA Health Department of Pediatrics, Charlottesville, Virginia, USA

## Abstract

Infantile Refsum disease is a rare peroxisomal biogenesis disorder characterized by impaired alpha-oxidation and accumulation of phytanic acid in the tissues. Patients often present with fundus changes resembling retinitis pigmentosa, developmental delay, sensorineural hearing loss, ataxia, and hepatomegaly. Traditionally, mainstay treatment for this condition has been a phytanic acid-restricted diet, although supplementation with either docosahexaenoic acid or cholic acid has rarely been described in the literature. We present a case of infantile Refsum disease in a child with retinitis pigmentosa-like ocular findings, sensorineural hearing loss, and self-resolving hepatic disease, who developed novel findings of macular edema refractory to carbonic anhydrase inhibitors. We describe management with a phytanic acid-restricted diet and combination docosahexaenoic acid, and cholic acid therapy, which helped to limit progression of her disease.

## 1. Introduction

Heredopathia Atactica Polyneuritiformis (Refsum disease) is a rare autosomal recessive disorder characterized by the accumulation of phytanic acid in the blood and tissues. A variant of this disease that occurs in children is infantile Refsum disease (IRD) which is biochemically, genetically, and clinically distinct from the adult form [1]. IRD is a mild Zellweger spectrum disorder characterized by retinitis pigmentosa-like fundi, developmental delay, sensorineural hearing loss, ataxia, and hepatomegaly [2]. The pathophysiology of IRD is driven by pathogenic variations in one of the 12 different genetic PEX genes that code for the biogenesis of peroxisomes [3]. This peroxisomal biogenesis abnormality leads to defective alpha-oxidation of dietary phytanic acid, a branched chain fatty acid present in meat, fish oils, and dairy products [3, 4]. The disorder is rare, although no exact estimates of IRD prevalence are available [5]. Low incidence and relatively nonspecific presentation may lead to a delay in diagnosis of the disease. We present the case of a five-year-old girl in whom the diagnosis of IRD was prompted by retinal pigmentary changes atypical for retinitis pigmentosa. Multimodal imaging of the retinal fundus is presented, documenting a novel finding of bilateral cystic macular edema (CME) in a patient with IRD. Early detection allowed systemic management of this patient with a phytanic acid-restricted diet and novel combination management with docosahexaenoic acid (DHA) and cholic acid.

## 2. Case Presentation

A 5-year-old girl with a clinical diagnosis of Usher syndrome was referred for initial retinal evaluation in our clinic. Her past ocular history was significant for left-sided esotropia treated with patching and atropine penalization of the dominant eye. She had a history of developmental delay, seizures, and significant hearing impairment. At the age of four months, she presented with severe hepatic dysfunction, with elevated serum levels of transaminases, and liver biopsy results were consistent with Alagille syndrome or a peroxisomal storage disorder. Previous workup included negative skin biopsy and no evidence of TORCH infection or renal dysfunction. Her enzyme levels improved spontaneously. The mother had a history of fetal demise at about 16 weeks of gestation secondary to cystic hygroma. The family history was otherwise unremarkable for congenital anomalies or known genetic disorders.

**Figure 1:**
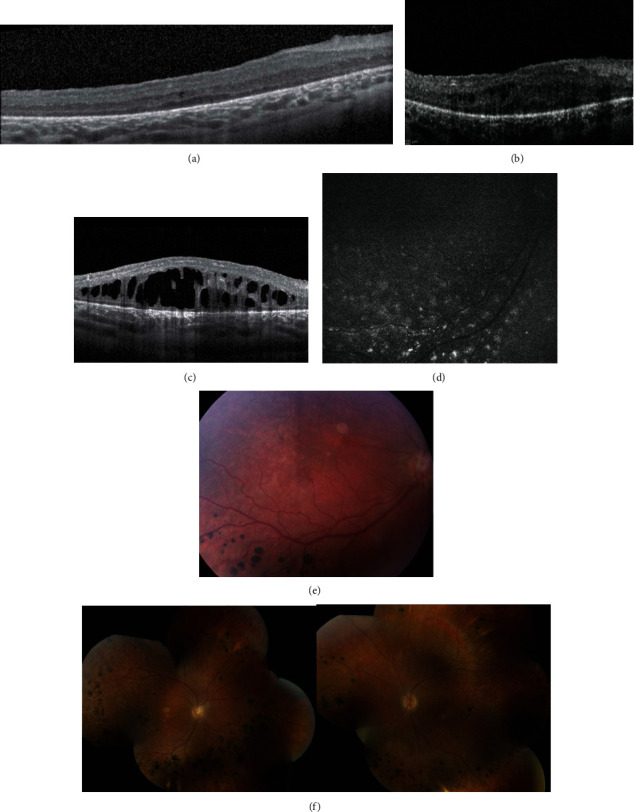
(a) OCT at age 5 on presentation of right eye showing minimal perifoveal cystic changes and extensive loss of IS/OS junction. (b) OCT of the right eye at 6 years of age demonstrating cystoid macular edema. Similar findings were noted in her left eye as well. (c) OCT of the right eye demonstrating persistent macular edema despite carbonic anhydrase inhibitors. Similar findings noted in the left eye. (d) Representative retinal autofluorescence revealed diffuse spotted pattern of hyperautofluorescence in midperiphery. (e) Central view of fundoscopic imaging revealing nummular patches. (f) Fundoscopic montage imaging at last follow-up revealing bilateral salt and pepper changes, a hypopigmented patch in the macula of both eyes, and bilateral nummular patches of hyperpigmentation in periphery. The optic nerve had no significant pallor, and the vessels were not attenuated.

Her physical exam upon our initial evaluation at age 5 years revealed the presence of epicanthal folds, upper lid ptosis, and bilateral single transverse palmar creases. She had approximately 15 degrees of residual left esotropia at presentation. Snellen visual acuity and intraocular pressure could not be obtained during the initial visit due to poor cooperation. The patient was able to fix and follow with each eye. Fundoscopic examination revealed bilateral “salt and pepper” changes, a hypopigmented patch in the macula of both eyes, and bilateral nummular patches of hyperpigmentation in the periphery, without significant ocular nerve pallor or vascular attenuation. OCT revealed few cystic changes in the macula with loss of foveal depression and ellipsoid zone in the right eye (Figure 1(a)).

Given the involvement of both the central and peripheral retinas with atypical hypopigmented nummular patches, the absence of disc pallor or significant vascular attenuation, and the absence of typical bone spicules, the previous diagnosis of Usher syndrome was questioned. Measurement of serum very long chain fatty acids showed elevation of C26:0 and C22:0 puritanic acid and phytanic acid, suggestive of a possible peroxisomal biogenesis disorder. Review of an electroretinogram (ERG) obtained under anesthesia at an outside facility revealed complete extinction of scotopic and photopic responses. Chromosomal microarray testing and Usher syndrome mutation panel were unremarkable. Genetic evaluation revealed that she was homozygous for a PEX1 mutation (c.2528 G>A, p. GLY843ASB) consistent with infantile Refsum disease.

Following the diagnosis, the patient was started on a phytanic acid-restricted diet and underwent audiology evaluation with subsequent hearing aid prescription for severe sensorineural hearing loss. At 6 years of age, she was noted to develop new bilateral cystoid macular edema, a finding not previously reported in this syndrome (Figure 1(b)). At the age of 9, she began therapy with 200 mg daily docosahexaenoic acid and 350 mg daily cholic acid (Cholbam). The patient's developmental delay improved on this treatment regimen, and the patient began excelling in schoolwork. However, despite long-term therapy with topical dorzolamide for 5 years, her macular edema persisted (Figure 1(c)) through her most recent follow-up at 13 years of age. At this time, 8 years after initial presentation, her Snellen visual acuity was 20/100 in the right eye and 1/200 E in the left. No cataracts were noted. Retinal autofluorescence revealed a diffuse spotted pattern of hyperautofluorescence in the midperiphery (Figure 1(d)). Fundus photos demonstrated stable bilateral salt and pepper changes, with bilateral nummular patches of hyperpigmentation with a hypopigmented patch in the macula of each eye (Figures 1(e) and 1(f)). The patient has some dental enamel irregularities but is currently doing well with no further complications due to her condition.

## 3. Discussion

This case highlights the unique clinical features that should prompt an investigation for systemic conditions in a pediatric patient with retinal findings mimicking retinitis pigmentosa. The diagnosis of IRD is challenging but can be life-changing, if made early, by preventing progression of the disease, developmental delay, and subsequent clinical sequelae. The diagnosis is complicated by the paucity of literature on IRD and confusion between the adult and infantile forms. The findings that prompted further testing and eventual diagnosis in this patient included her young age and atypical nummular pigmented patches without significant optic nerve pallor or vascular attenuation. It is interesting that the patient's earlier liver abnormalities had stabilized without specific treatments following a genetic diagnosis in infancy. Successful diagnosis allowed our patient to receive treatment with phytanic acid diet restriction, docosahexaenoic acid, and cholic acid and has allowed for normal cognitive development, thus far no additional systemic manifestations of the disease.

IRD is rare, and its clinical characteristics have not been fully elucidated [5]. In contrast to classic Refsum disease, the infantile form is associated with minor dysmorphism, mental retardation, hepatomegaly, and sensorineural hearing loss [2]. Within the limited literature, retinal pigmentary degeneration and accompanying night blindness are commonly reported as earliest ocular clinical manifestations of IRD [6]. These ocular findings are similar to that of an inherited retinal dystrophy, which can be seen in a number of syndromes such as Bardet-Biedl [7]. However, the nummular patches of hyperpigmentation seen in our patient are not typical for an inherited dystrophy. Other nonspecific findings such as “salt and pepper” spots and nystagmus are also found in a variety of other conditions, but the patient's elevated phytanic acid is most suggestive of peroxisomal disease. As far as we are aware, this is the first report of a patient with IRD with cystoid macular edema. Furthermore, her CME was found to be refractory to treatment with topical carbonic anhydrase inhibitor.

The pathophysiology of IRD is driven by defective alpha oxidation of phytanic acid and other related structures derived from common dietary sources [8–10]. Clinical symptoms arise when excess phytanic acid accumulates in nervous tissues and liver [3–6, 11]. Consequently, detection and early management of IRD is critical in preventing irreversible long-term sensorineural degeneration [12]. Sequelae may include ataxia secondary to cerebellar degeneration, seizures, and even death from nerve fibrosis and fluid accumulation within the myelin sheath of critical nerves [13–15]. Vision loss also occurs secondary to deposition of excess phytanic acid within ocular tissue, manifesting as photoreceptor and ganglion cell death primarily in peripheral fields [16].

The specific PEX1 pathogenic variation found in this patient (G843D) has previously been postulated in the literature to account for up to one-third of all pathogenic PEX1 gene alleles [16]. This variant has been shown to follow an autosomal recessive inheritance pattern though this was not confirmed with parental testing in our case. While functional studies analyzing the predicted effect on the PEX1 protein is lacking, it is believed to impact interactions with the PTS1 protein. However, one expression study assaying the G843D PEX1 allele's effect on PTS1 protein import demonstrated up to 15% residual catalase import activity compared to wild-type PEX1, suggesting that the G843D variant portends a more mild clinical phenotype compared other known PEX1 variants [16, 17].

Traditionally, the main treatment modality for patients with IRD has been centered around a phytanic acid-free diet. While it is possible for phytanic acid to be metabolized from adipose tissue, it predominantly comes from dietary sources [18]. Plasma exchange therapy has also been proposed to remove phytanic acid directly from the blood and has been demonstrated to improve patient outcomes when dietary restriction is insufficient in managing plasma phytanic acid. Though excluding phytanic acid from the diet has not been shown to reverse optic neuropathy [5], it can alleviate systemic complications and drastically improve quality of life [18]. However, phytanic acid-free foods are not palatable, leading to lower patient compliance with dietary regiments [19].

Our patient has benefitted from hearing aids, a switch to a vegan diet, and adjunctive treatment with a combination of docosahexaenoic acid and cholic acid, which appeared to slow progression of her IRD. To our knowledge, this is the first reported use of docosahexaenoic acid and cholic acid in combination for IRD. However, these drugs have been used individually in patients with Zellweger spectrum disorders with controversial efficacy. These drugs are aimed at increasing deficient polyunsaturated fatty acids in patients with Zellweger spectrum disorders and decreasing the concentrations of toxic bile acid intermediates [20, 21]. While some studies suggest a beneficial effect of DHA supplementation in patients with Zellweger spectrum disorders, a randomized double-blind placebo-controlled trial showed no improvement of visual function and growth [21, 22]. Similarly, despite FDA approval, studies surrounding cholic acid supplementation have not demonstrated improved growth and development or reduced urinary excretion of toxic intermediates in these patients [23, 24]. Additional research is needed to evaluate efficacy of DHA and cholic acid as combination therapy for IRD.

In summary, it is important to rule out the diagnosis of IRD or other inborn errors of metabolism in any young patient presenting with pigmentary retinopathy, as early detection is paramount for reducing morbidity and mortality of these conditions. Cystoid macular edema has not previously been reported but appears to be an important cause of visual decline in patients with IRD and may progress over time despite treatment with carbonic anhydrase inhibitors as seen in our patient. Combination therapy with docosahexaenoic acid (DHA), cholic acid, and dietary restriction when introduced at a young age may offer patients a chance at near normal development and life.

## Abbreviations

DHAL: Docosahexaenoic acid
CME: Cystic macular edema
IRD: Infantile Refsum disease
OCT: Optical coherence tomography.
